# Loss of Coxsackie and adenovirus receptor downregulates *α*-catenin expression

**DOI:** 10.1038/sj.bjc.6605331

**Published:** 2009-09-22

**Authors:** K Stecker, A Koschel, B Wiedenmann, M Anders

**Affiliations:** 1 Division of Gastroenterology and Hepatology, Department of Internal Medicine, Charité Medical School, Campus Virchow, Augustenburgerplatz 1, Berlin 13353, Germany; 2 Department of Interdisciplinary Endoscopy, University Hospital Hamburg Eppendorf, Martinistraße 52, Hamburg 20246, Germany

**Keywords:** Coxsackie and adenovirus receptor, *α*-catenin, interaction

## Abstract

**Background:**

The Coxsackie and adenovirus receptor (CAR) has been shown to inhibit cancer cell proliferation, migration, and invasion. The underlying mechanisms, however, are poorly understood.

**Methods:**

The differential gene expression in the human colon cancer cell line DLD1 on RNAi-mediated functional CAR knockdown was analysed using oligo-array technology. Expression of *α*-catenin was determined by quantitative RT-PCR and western blotting. Proliferation, migration, and invasion after CAR knockdown were assessed by *in vitro* assays, and cell morphology in a three-dimensional context was evaluated using matrigel.

**Results:**

Oligo-array technology identified *α*-catenin as the strongest downregulated gene after CAR knockdown. Western blotting and quantitative RT-PCR confirmed a reduced *α*-catenin expression after CAR knockdown in DLD1 cells and in the rat intestinal cell line IEC-6. Functionally, both cell lines showed a marked increase in proliferation, migration, and invasion on CAR knockdown. In matrigel, both cell lines formed amorphous cell clusters in contrast to well-organised three-dimensional structures of CAR-expressing vector controls. Ectopic ‘re’-expression of *α*-catenin in DLD1 and IEC-6 CAR knockdown cells reversed these functional and morphological effects.

**Conclusion:**

These data suggest that an interaction of CAR and *α*-catenin mediates the impact of CAR on cell proliferation, migration, invasion, and morphology.

The Coxsackie and adenovirus receptor (CAR) was first characterised as a viral attachment site on the surface of epithelial cells and was later identified as an integral component of the tight junction complex (Coyne and Bergelson, 2005). In several human carcinomas, particularly in more advanced stages, a reduced CAR presence was found, partially associated with loss of tumour differentiation, increased infiltration, and poor prognosis (Sachs *et al*, 2002; Matsumoto *et al*, 2005; Korn *et al*, 2006; Buscarini *et al*, 2007; Okegawa *et al*, 2007; Anders *et al*, 2009). On the basis of data from studies in cell lines, it has been speculated that loss of CAR promotes the proliferation, migration, and invasion of cancer cells (Okegawa *et al*, 2000, 2001; Bruning and Runnebaum, 2003, 2004; Huang *et al*, 2005; Wang *et al*, 2005). These phenomena are believed to result from an impaired intercellular adhesion (Okegawa *et al*, 2000; Bruning and Runnebaum, 2004; Wang *et al*, 2005). Recently, it has been shown that CAR interacts with microtubules, and therefore contributes to the cytoskeletal equilibrium and inhibits cell migration (Fok *et al*, 2007). A more detailed insight into the mechanisms underlying the influence of CAR on cancer cell pathobiology, however, is lacking.

Aiming to further elucidate genes that facilitate the proposed function of CAR in cancer, we determined differential gene expression after CAR knockdown in the human colon cancer cell line DLD1 and assessed the functional impact of the strongest regulated gene *α*-catenin.

## Materials and methods

### Cell culture and generation of transfected cell lines

The human colon cancer cell line DLD1 and the rat small intestine cell line IEC-6 were obtained from the American Type Culture Collection (Rockville, MD, USA) and from the Deutsche Sammlung von Mikroorganismen und Zellkulturen GmbH (Braunschweig, Germany), respectively, and were cultured in recommended growth media. Three-dimensional cultures were set up as described previously (Anders *et al*, 2003b). In brief, cell lines were cultured to confluence as monolayers and subsequently embedded into a commercially prepared reconstituted basement membrane from Engelbreth–Holm–Swarm tumours (BD Biosciences, Bedford, MA, USA). Cell lines with a functional CAR knockdown using CAR-specific siRNAs were generated as described previously (Anders *et al*, 2009). In this study, either a CAR-specific siRNA, CCAAGUACCAAGUGAAGACdTdT, or a control siRNA, CACAAAAGUAUCGCGCAAGdTdT, cloned into the ‘pSuper’ vector system (Oligoengine, Seattle, WA, USA) was transfected into cell lines using Effectene (Quiagen, Hilden, Germany). The ectopic expression of *α*-catenin was accomplished by the stable transfection of a human full-length *α*-catenin cDNA (a kind gift from M Ozawa, Kagoshima University, Kagoshima, Japan) as described previously (Matsubara and Ozawa, 2004). The differential expression of CAR and the *α*-catenin protein in pooled cell populations was determined by western blotting.

### Oligo-array analysis

Total RNA was isolated using Trizol (Invitrogen, Karlsruhe, Germany) and reverse transcribed with oligo-dT primers and SuperScript II (Invitrogen). cDNAs generated from 200 ng of total RNA were analysed for differential gene expression using HG U133 2.0 plus chips (Affymetrix, Santa Clara, CA, USA) and processed using GeneSpring software (Agilent Technologies, Inc., Palo Alto, CA, USA).

### Western blotting

Protein lysates were obtained as previously described (Anders *et al*, 2003a). Equal amounts of protein lysates were loaded onto reducing Laemmli gels, immunoblotted with specific antibodies against CAR (H-300: sc-15405; Santa Cruz Biotechnology, Santa Cruz, CA, USA), *α*-catenin, ZO-1 (both Zymed Laboratories, South San Francisco, CA, USA), or *β*-Actin (Sigma-Aldrich, Munich, Germany), and detected using the enhanced chemiluminescence system (Amersham Pharmacia, Piscataway, NJ, USA).

### Quantitative mRNA determination

Total RNA was isolated using Trizol (Invitrogen) and was reverse transcribed with oligo-dT primers and SuperScript II (Invitrogen). cDNAs generated from 200 ng of total RNA were used for real-time RT-PCR assays on a Stratagene MX3000P cycler using *α-*catenin gene-specific primers (CCATGCAGGCAACATAAACTTC and AGGGTTGTAACCTGTGTAACAAG) and the qPCR MasterMix for SYBR Green I (Eurogentec Deutschland GmbH, Köln, Germany). Quantification was carried out by the comparative Δ*C*_T_ method normalising *C*_T_ values to *β*-actin (DLD1) or 18S (IEC-6). cDNAs derived from CHO and CHO-CAR cells were used as negative and positive controls, respectively. All experiments were carried out in triplicate and repeated at least twice.

### Immunofluorescence

Immunofluorescence staining was carried out as described previously (Anders *et al*, 2003a). In brief, anti-CAR (H-300: sc-15405; Santa Cruz Biotechnology) and anti-*α*-catenin (Zymed Laboratories) antibodies served as primary antibodies; a Cy2-conjugated anti-rabbit antibody and a Cy3-conjugated anti-mouse antibody were used as secondary antibodies (Dianova GmbH, Hamburg, Germany). Multicolour fluorescence microscopy was carried out using a Zeiss Axiophot microscope (Carl Zeiss AG, Jena, Germany).

### Co-immunoprecipitation

Co-immunoprecipitations were carried out using the Immunoprecipitation Kit Protein G (Roche Diagnostics GmbH, Mannheim, Germany) according to the manufacturer's instructions. In brief, anti-CAR (H-300: sc-15405; Santa Cruz Biotechnology) and anti-*α*-catenin (Zymed Laboratories) antibodies served as antibodies for the initial immunoprecipitation; IgG rabbit (Dianova GmbH) was used as control. Subsequent western blotting was carried out as described above.

### Assessment of cell proliferation

Cells were seeded onto six-well plates (4 × 10^5^ cells per well for DLD1 and 2 × 10^5^ cells per well for IEC-6) in recommended media. After 48 h, the cells were detached using trypsin, stained with trypan blue to exclude dead cells, and counted using a haematocytometer counting chamber (VWR International, Darmstadt, Germany). All experiments were carried out in triplicate and repeated at least twice.

### Determination of cell migration

Assessment of directed cancer cell migration was carried out using an AP48 48 Well Micro Chemotaxis Chamber (Neuro Probe, Gaithersburg, MD, USA). Here, 50 000 cells per well in 50 *μ*l fetal calf serum (FCS)-free media were seeded onto the upper part of the chamber, whereas its lower compartment was filled with media containing 10% FCS or serum-free media as control. After 24 h at 37°C, cells that migrated through the filter were fixed and stained with crystal violet containing 10% ethanol. After gentle rinsing with phosphate-buffered saline, non-migrated cells at the upper side of the filter were scraped off. Subsequently, the crystal violet dye retained on the filters was extracted with 10% acetic acid for 5 min at room temperature and was colorimetrically measured at 560 nm. All experiments were carried out in triplicate and repeated at least twice.

### Cell invasion into matrigel

Cells were seeded onto the top of BioCoat Matrigel Invasion Chambers (BD Biosciences) containing 8 *μ*m pore size PET membranes covered with matrigel matrix. Medium containing 10% FCS was added to the bottom well of the chambers as a chemoattractant, whereas serum-free medium was used as a control. After 24 h at 37°C and 5% CO_2_, cells that invaded the matrigel-coated membrane, located at the lower membrane surface, were fixed and stained by crystal violet containing 10% ethanol. Cells of three representative areas in each well were counted at a magnification of × 100. Experiments were carried out in triplicate and repeated at least twice.

### Statistical analysis

Statistical calculations were carried out using GraphPad Prism software (version 4.00; GraphPad Software, Inc., San Diego, CA, USA) using an unpaired, two-tailed *t*-test, considered significant when *P*<0.05.

## Results

### Downregulation of CAR induces loss of *α*-catenin

**Figure 1:**
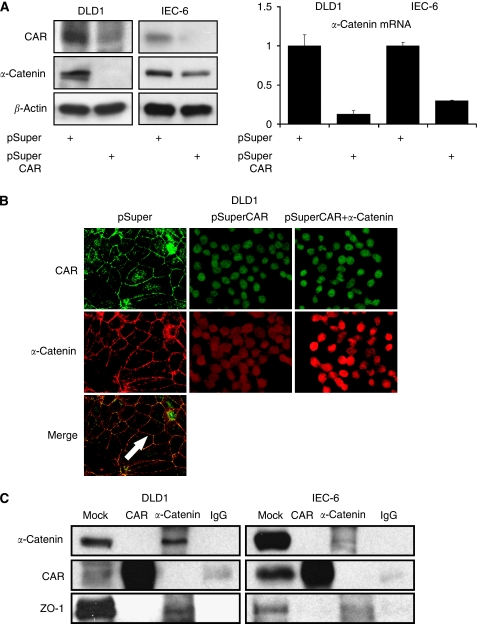
Expression and localisation of CAR and *α*-catenin. Protein expression levels of CAR, *α*-catenin, and *β*-actin were analysed by western blotting (**A**, left panel). Transcriptional regulation of *α*-catenin after CAR downregulation shown by qRT-PCR with expression levels in mock vector-transfected controls set to 1 (**A**, right panel). Immunofluorescence co-staining for CAR and *α*-catenin reveals the presence of both proteins at the plasma membrane (arrow) in mock vector-transfected controls (left panels), loss of protein presence at the plasma membrane after CAR downregulation (intracellular signal intensity equals controls that contain secondary antibodies only) (middle panels), and intracellular immunopositivity for *α*-catenin after ‘re’-expression of *α*-catenin (right panels) (magnification × 630) (**B**). Co-immunoprecipitations indicate no binding between CAR and *α*-catenin, yet confirm an interaction of *α*-catenin and ZO-1 (**C**).

Gene expression profiles of DLD1 cells after siRNA-mediated CAR knockdown (DLD1^CAR-negative^) were compared with those of DLD1^control^ cells (containing the pSuper vector only), using oligo-array technology. Setting a threshold of a 1.5-fold difference between the two cell lines, 1187 genes were found upregulated and 2318 genes downregulated in the DLD1^CAR-negative^ cell line. Among these genes, *α*-catenin displayed the highest fold change, showing an 18.54-fold lower expression in the DLD1^CAR-negative^ cell line. Western blotting confirmed this finding at the protein level revealing a reduced *α*-catenin expression in both the DLD1^CAR-negative^ and the IEC-6^CAR-negative^ cell lines. Furthermore, transcriptional dependence of *α*-catenin by CAR was shown using reverse transcribed (RT)–PCR assays showing a reduced mRNA expression level of *α*-catenin in DLD1^CAR-negative^ and IEC-6^CAR-negative^ cells (Figure 1A).

### CAR and *α*-catenin co-localise, yet do not bind directly

Co-immunofluorescence staining for CAR and *α*-catenin revealed a ‘honeycomb’ pattern of immunopositivity at the plasma membrane showing a co-localisation of both proteins at this site in parental- (data not shown) and vector-transfected control cell lines. In contrast, DLD1^CAR-negative^ and IEC-6^CAR-negative^ cells showed a weak intracellular signal only, comparable with that of controls stained by secondary antibodies only (data not shown). After the ectopic expression of *α*-catenin in DLD1^CAR-negative^ and IEC-6^CAR-negative^ cell lines, a strong intracellular immunopositivity was noted for *α*-catenin, yet no signal was detected at the plasma membrane (Figure 1B). To elucidate whether CAR and *α*-catenin proteins bind to each other, we performed co-immunoprecipitations. These analyses showed binding neither when carried out with the anti-CAR antibody nor when carried out with the anti-*α*-catenin antibody. To test whether our experimental conditions are sufficient to reveal inter-protein binding, additional western blotting was carried out with an anti-ZO-1 antibody, a known binding partner of *α*-catenin (Imamura *et al*, 1999). These experiments confirmed the binding of ZO-1 to *α*-catenin in both cell lines (Figure 1C).

### Impact of CAR and *α*-catenin on cellular morphology

**Figure 2:**
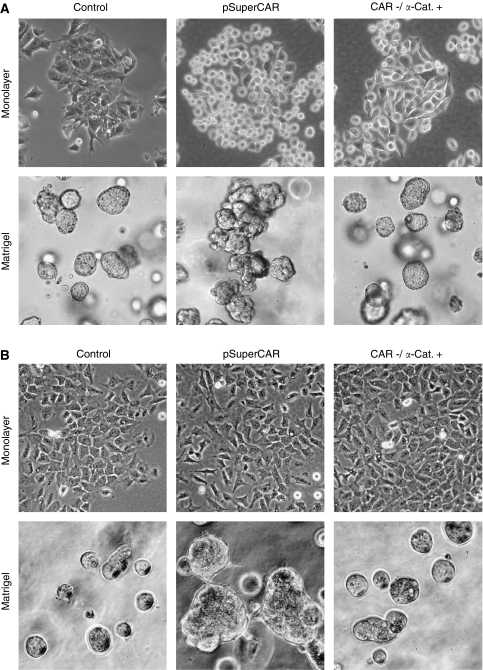
Impact of CAR and *α*-catenin on cellular morphology. Cells after CAR knockdown appear round and smaller compared with controls (DLD1) or display less intercellular attachment sites (IEC-6). In matrigel, cells after CAR knockdown form amorphous clusters in contrast to the organised-appearing formations of matching controls. Ectopic ‘re’-expression of *α*-catenin partially reverses the effects seen after CAR knockdown when grown as a monolayer, and particularly results in organised cell formations similar to those of vector controls when cultured in matrigel. Images show representative results for DLD1 (**A**) and IEC-6 (**B**).

When cultured under conventional conditions, slight changes in cell shape were noted for both cell lines: DLD1^CAR-negative^ cells appeared rounder and smaller compared with controls, whereas IEC-6^CAR-negative^ cells showed a spindlier shape with less intercellular contact. More dramatic differences were observed when cells were cultured in matrigel, enabling cells to form three-dimensional structures. Both cell lines formed amorphous clusters in sharp contrast to the well-organised-appearing formations of control cell lines. The ectopic ‘re’-expression of *α*-catenin in DLD1^CAR-negative^ and IEC-6^CAR-negative^ partially reversed the effects of CAR knockdown, resulting in bigger-, flatter-, and angular-appearing cells. When grown in matrigel, these cell lines established organised formations similar to those of DLD1^control^ and IEC-6^control^ (Figure 2).

### Impact of CAR on cell proliferation, migration, and invasion is mediated by *α*-catenin

**Figure 3:**
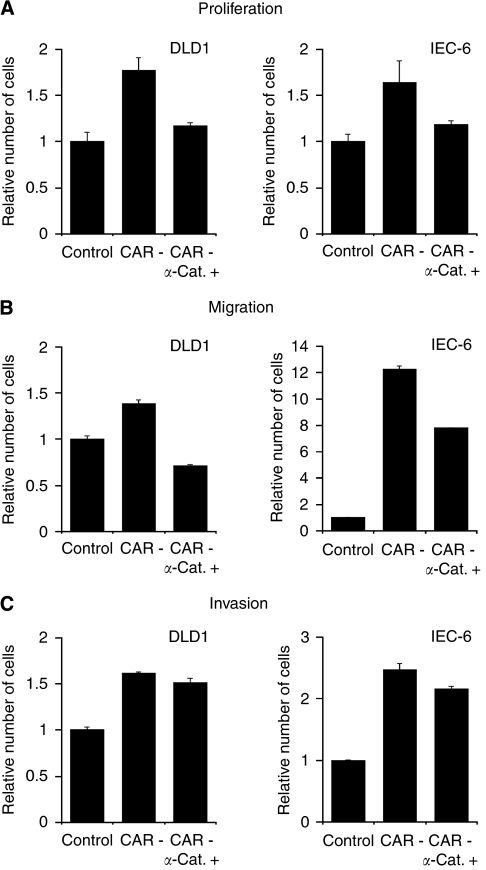
Functional impact of CAR downregulation and *α*-catenin ‘re’-expression in DLD1 and IEC-6 cell lines. Proliferation (**A**), migration (**B**), and invasion (**C**) in DLD1 and IEC-6 were determined after CAR knockdown and ‘re’-expression of *α*-catenin in comparison with controls.

To clarify the functional influence of the CAR–*α*-catenin interaction, we performed *in vitro* assays after CAR knockdown and the ectopic ‘re’-expression of *α*-catenin. Assessment of cell proliferation revealed significantly higher cell numbers in DLD1^CAR-negative^ and IEC-6^CAR-negative^ compared with controls upon 48 h of cultivation (DLD1^CAR-negative^ *P*=0.0017; IEC-6^CAR-negative^ *P*=0.0129). Ectopic ‘re’-expression of *α*-catenin in DLD1^CAR-negative^ and IEC-6^CAR-negative^ resulted in significantly lower cell numbers (DLD1^CAR-negative/*α*-catenin^ *P*=0.0022; IEC-6^CAR-negative/*α*-catenin^ *P*=0.0319) (Figure 3A). Using migration assays, DLD1^CAR-negative^ and IEC-6^CAR-negative^ showed significantly increased migratory properties compared with vector-control cell lines (DLD1^CAR-negative^ *P*=0.0005; IEC-6^CAR-negative^ *P*<0.0001). In contrast, ectopic ‘re’-expression of *α*-catenin significantly decreased the number of migrating cells for DLD1^CAR-negative/*α*-catenin^ and IEC-6^CAR-negative/*α*-catenin^ (both: *P*<0.0001) (Figure 3B). To test whether cells migrate in an FCS-directed manner, we included FCS-free medium controls for each of the tested cell lines, without noting cell migration in any case (data not shown). The number of cells invading into matrigel compared with vector controls increased significantly for both DLD1^CAR-negative^ (*P*<0.0001) and IEC-6^CAR-negative^ (*P*<0.0001) cell lines. Ectopic ‘re’-expression of *α*-catenin impaired cell invasion significantly (DLD1^CAR-negative/*α*-catenin^ *P*=0.0318; IEC-6^CAR-negative/*α*-catenin^ *P*=0.0129), yet was not sufficient to reduce the number of invading cells to the level of DLD1^control^ and IEC-6^control^ cell lines (Figure 3C).

## Discussion

This study marks the first report of an impaired *α*-catenin expression after downregulation of CAR. On the basis of our data, it may be speculated that this phenomena crucially affects the inhibitory effect of CAR on cell proliferation, migration, and invasion. Moreover, our observations reveal that loss of CAR is potentially accompanied by substantial changes in cellular morphology that may also be explained by an impaired presence of *α*-catenin.

Our findings reveal that a reduced *α*-catenin mRNA expression represents the most prominent feature after downregulation of CAR. This observation was further validated by our finding of reduced *α*-catenin protein presence in DLD1 and IEC-6 cell lines upon downregulation of CAR. As DLD1 represents a human cancer cell line and IEC-6 a rat non-transformed small intestinal cell line, it may be speculated that our results account for a more general phenomenon. However, differential protein expression of *α*-catenin after CAR downregulation differs between cell lines: after a transient transfection of a CAR-specific siRNA, reduced *α*-catenin protein expression was noted in AGS (gastric cancer) and SW480 (colon cancer), whereas in T84 and HCT116 cells, only a moderate decline was observed and no changes were noted in Caco2 (all colon cancer; data not shown). The underlying mechanisms of these differences remain to be elucidated. Transcriptional regulation of *α*-catenin expression after CAR downregulation potentially marks the main regulatory measure, as shown by quantitative RT-PCR. However, whether the *α*-catenin promoter becomes inactivated upon loss of CAR or changes in RNA stability occur, remains to be elucidated.

The previously proposed inhibitory effects of CAR on cancer cell growth and motility have been suggested to result from an impaired intercellular adhesion (Okegawa *et al*, 2000; Bruning and Runnebaum, 2004; Wang *et al*, 2005). Our data show that loss of CAR impairs the expression of *α*-catenin, a protein that has been extensively studied for its inhibition of cancer cell growth and motility (Scott and Yap, 2006). Moreover, loss of *α*-catenin is frequently described in carcinomas, for example, colon cancer, significantly correlating with depth of invasion, presence of metastasis, and an adverse clinical outcome (Ropponen *et al*, 1999). In line with these reports, we noted an increased proliferation, migration, and invasion after loss of CAR and *α*-catenin. Furthermore, our data show that ‘re’-expression of *α*-catenin is sufficient to markedly reverse these effects. Therefore, it may be speculated that the functional effects on cell growth and motility after loss of CAR are crucially mediated by a reduced expression of *α*-catenin. However, cell invasion has been reduced to a small extent only on re-expression of *α*-catenin. Given that our experimental setting sufficiently reflects cell invasion, we found that a CAR–*α*-catenin interaction seems to be less important in this context compared with cell proliferation and migration.

Both *α*-catenin and, recently, CAR have been shown to interact with the cytoskeleton (Scott and Yap, 2006; Fok *et al*, 2007). In agreement with these studies, we noted substantial changes in cell morphology, particularly when using three-dimensional culturing, of cell lines after CAR knockdown. The impact of *α*-catenin on this phenomenon was shown by the ‘re-’expression of *α*-catenin, which resulted in the formation of organised cell formations as opposed to amorphous clusters after CAR knockdown.

Co-immunofluorescence staining for CAR and *α*-catenin showed a co-localisation of both proteins at the plasma membrane in parental- and vector-transfected control cell lines. However, the ‘re’-expression of *α*-catenin in CAR-negative cell lines resulted in an intracellular accumulation of *α*-catenin, yet no ‘re’-distribution to the plasma membrane. Therefore, we hypothesised that CAR may recruit *α*-catenin to the plasma membrane. Yet, co-immunoprecipitation using either an anti-CAR or an anti-*α*-catenin antibody failed to proof a binding of these proteins. Therefore, reduced presence of other proteins that link *α*-catenin to the plasma membrane may account for the intracellular accumulation of *α*-catenin seen here. Nevertheless, binding of CAR to *α*-catenin may not be excluded entirely, as it might be weak and/or transient and not be detectable biochemically. Herein, our findings are potentially in line with a recent study showing that *α*-catenin, rather than forming a stable link to actin, acts as a regulator of actin dynamics and that its binding to the E-cadherin–*β*-catenin complex is transient and mutually exclusive of its interaction with F-actin (Drees *et al*, 2005).

In conclusion, it may be speculated that the suggested tumour suppressive function of CAR is at least in part mediated by regulating the *α*-catenin expression. Underlying regulatory mechanisms remain to be elucidated.
